# Quality of care for remote orthopaedic consultations using telemedicine: a randomised controlled trial

**DOI:** 10.1186/s12913-016-1717-7

**Published:** 2016-09-08

**Authors:** Astrid Buvik, Einar Bugge, Gunnar Knutsen, Arvid Småbrekke, Tom Wilsgaard

**Affiliations:** 1 Department of Orthopaedic Surgery, University Hospital of North Norway, P.O. Box 4, N-9038 Tromsø, Norway; 2 Centre for Clinical Research and Education, University Hospital of North Norway, P.O. Box 20, N-9038 Tromsø, Norway; 3 Department of Community Medicine, Faculty of Health Sciences, UiT The Arctic University of Norway, 9037 Tromsø, Norway

**Keywords:** Telemedicine, Videoconference, Orthopaedic, Outpatient clinic consultation, Randomised, Physicians, Safety, Evaluation

## Abstract

**Background:**

Decentralised services using outreach clinics or modern technology are methods to reduce both patient transports and costs to the healthcare system. Telemedicine consultations via videoconference are one such modality. Before new technologies are implemented, it is important to investigate both the quality of care given and the economic impact from the use of this new technology. The aim of this clinical trial was to study the quality of planned remote orthopaedic consultations by help of videoconference.

**Method:**

We performed a randomised controlled trial (RCT) with two parallel groups: video-assisted remote consultations at a regional medical centre (RMC) as an intervention versus standard consultation in the orthopaedic outpatient clinic at the University Hospital of North Norway (UNN) as a control. The participants were patients referred to or scheduled for a consultation at the orthopaedic outpatient clinic. The orthopaedic surgeons evaluated each consultation they performed by completing a questionnaire. The primary outcome measurement was the difference in the sum score calculated from this questionnaire, which was evaluated by the non-inferiority of the intervention group. The study design was based on the intention to treat principle. Ancillary analyses regarding complications, the number of consultations per patient, operations, patients who were referred again and the duration of consultations were performed.

**Results:**

Four-hundred patients were web-based randomised. Of these, 199 (98 %) underwent remote consultation and 190 (95 %) underwent standard consultation. The primary outcome, the sum score of the specialist evaluation, was significantly lower (i.e. ‘better’) at UNN compared to RMC (1.72 versus 1.82, *p* = 0.0030). The 90 % confidence interval (CI) for the difference in score (0.05, 0.17) was within the non-inferiority margin. The orthopaedic surgeons involved evaluated 98 % of the video-assisted consultations as ‘good’ or ‘very good’. In the ancillary analyses, there was no significant difference between the two groups.

**Conclusions:**

This study supports the argument that it is safe to offer video-assisted consultations for selected orthopaedic patients. We did not find any serious events related to the mode of consultation. Further assessments of the economic aspects and patient satisfaction are needed before we can recommend its wider application.

**Trial registration:**

ClinicalTrials.gov identifier: NCT00616837

**Electronic supplementary material:**

The online version of this article (doi:10.1186/s12913-016-1717-7) contains supplementary material, which is available to authorized users.

## Background

Patients need secondary care consultations after referrals from their general practitioners (GPs), or they need follow-up consultations for earlier treatment or for chronic disease. According to the health authorities in Norway, it is a public responsibility to provide necessary health and care services to the entire population regardless of place of residence. Decentralised services using outreach clinics or modern technology are methods to reduce both patient transports and costs to the healthcare system [1]. The University Hospital of North Norway (UNN) is the tertiary referral hospital for the North Norway regional health trust, covering approximately 470,000 inhabitants (2012) and an area of 112,975 km^2^. UNN is also the local hospital for Troms and northern Nordland County, covering 187,000 inhabitants (2012) and an area of 31,500 km^2^. In 2014, the trust’s expenses for patient travel accounted for 3.2 % of the hospital’s total budget, not including expenses for ambulance transport by car, boat or air [2]. As one of the outpatient clinics with the highest number of patients, many of whom need assistance by accompanying persons when travelling or who are not able to use public transport, decentralising orthopaedic outpatient consultations is of special interest.

Telemedicine equipment is improving rapidly with regard to quality, cost and user-friendliness; these, together with the distribution of high-speed telecommunication networks, may make it tempting to implement this new technology without further investigation. However, before new methods in healthcare delivery are implemented, it is important to investigate the quality and safety of the care given as well as the economic impact of such innovation to discover any pitfalls and reduce unwanted events. An earlier non-randomised study demonstrated good accuracy by telemedicine-assisted consultation for trauma management compared to standard consultations [3]. A randomised controlled trial (RCT) found telemedicine capable of providing a satisfactory standard of care in the management of minor injuries [4]. Another RCT evaluated patients coming to an emergency department and found telemedicine to be a satisfactory treatment technique [5]. Others suggest that telemedicine is an alternative to conventional visits for orthopaedic patients in an outpatient setting [6–9]. In one study, real-time videoconference was found to suitably provide orthopaedic care to rural areas; however, further investigations, including a cost–benefit analysis, were recommended [10]. Also, telehealth via real-time videoconference was reported to be effective by connecting an Antarctic station and Japan to treat orthopaedic cases [11]. However, there are few randomised studies regarding telemedicine and orthopaedic patients, none of which were conducted in Norway [12–16]. Some of the earlier studies demonstrated the importance of transmitting X-ray images of adequate quality as a factor to improve telemedicine for remote orthopaedic consultation; this was performed with a separate document camera [8, 17]. The X-ray system at UNN is fully digitalised: digital images taken at one location are electronically available at other locations within the hospital trust.

From this background, the aim of this RCT was to study the quality of remote telemedicine consultations in an outpatient clinic as compared to ordinary consultations. The study is reported according to the consort 2010 guidelines [18]. Telemedicine in this study means the use of real-time videoconference and digitalised X-rays. Our study hypothesis was as follows: The introduction of telemedicine service in the form of real-time videoconference for the selected orthopaedic patients will cause no reduction of the professional quality of the patient treatment administered by the doctor involved in the consultations; it will also increase patient satisfaction and lower costs. The study hypothesis examines the non-inferiority of telemedicine consultation versus conventional outpatient consultation. Our choice of a non-inferiority trial design was based on the expectation that a slightly lower-quality score of the evaluation by the physician of the video-assisted consultations would be compensated by increased patient satisfaction and/or reduced travel expenses. In this paper, we present the method of the study and the analyses of the professional quality of the patients’ treatment.

## Methods

This RCT featured two parallel groups that were allocated into remote consultations at a regional medical centre (RMC) (3.5 h by car from Tromsø) as an intervention and into standard consultations in the orthopaedic outpatient clinic at UNN as a control.

### Technical equipment

At RMC, a screen (ViewSonic, Modl nr VS10946-Ie) with a codec and camera situated on top (Tandberg 990MXP) was installed. The orthopaedic surgeon at UNN controlled the camera, which could be used to zoom in on the patients (to look at a post-operative wound) or follow them when walking, for example. At UNN, in a standard outpatient clinic room, another camera, codec (Tandberg 1500MXP) and similar screen were installed. These were connected to a standard PC to show the X-rays to the patient if he or she wanted. The Norwegian Health Network transmitted data over a secure broadband connection (10 Mbps full duplex).

### Participants

All of the patients were recruited from the four northernmost municipalities in Troms County in Northern Norway: Kåfjord, Skjervøy, Nordreisa and Kvænangen. The 6,500 km^2^ area is sparsely populated with approximately 12,000 inhabitants (in 2013), 50 % of which live in five small towns. The patients, who all were referred to or scheduled a visit at the orthopaedic outpatient clinic at UNN Tromsø, were evaluated according to the inclusion and exclusion criteria defined by the orthopaedists running the study (Table 1).

**Table 1:** Patients’ inclusion and exclusion criteria

Inclusion criteria	Exclusion criteria
New referred to orthopaedic outpatient clinic UNN, Tromsø (e.g. knee osteoarthritis, hallux valgus) Follow up after orthopaedic surgery (e.g. arthroplasty of the hip) Follow up after orthopaedic trauma (operated or not) Follow up of chronic orthopaedic disorders Written consent	Expectancy of advanced physical examination/tests (e.g. shoulder- and “young knee” problem) Unable to give informed consent (e.g. Dementia, soldiers, prisoners) Need of interpreter To be seen by a specific orthopaedic surgeon Need of contemporary procedures (e.g. CAT-scan, ultrasound) Contemporary other outpatient clinical consultation

### Interventions

The remote consultations were performed through real-time videoconference, where a trained nurse was with the patient at the remote location and the orthopaedic surgeon was located at UNN. The preselected orthopaedic surgeons (three consultants, two experienced registrars) carried out their daily work at the orthopaedic department and conducted the consultations as part of their daily routine. They were randomly selected according to who were available at the consultation time. The orthopaedic surgeon ran the consultation after some initial training and technical assistance. Before beginning the study, two nurses from the RMC were trained at the orthopaedic outpatient clinic. They attended casting courses and were trained in clinical examination techniques. The trained nurses received the patient at the remote site, assisted during the consultation and performed physical tasks, for example, changed a cast or removed stitches. No physician was with the patient at the remote site. A digital X-ray lab served by a radiograph was available at the RMC. Digital X-rays were, if appropriate, available at the time of the consultation. Radiologists at UNN later described the X-rays and included them in the hospital’s standard X-ray records. The standard consultations took place at the hospital outpatient clinic. In each consultation, the usual mandatory registration and documentation in the patient’s medical records was done by the orthopaedic surgeon, including the conclusion of the consultation, agreement between surgeon and patient regarding any follow-up appointments, prescriptions, referrals for operation, further investigation, physiotherapist training and/or an application for orthopaedic aid if needed.

### Outcomes

Following each consultation, the orthopaedic surgeon immediately evaluated the professional quality of the telemedicine and the standard consultation. The evaluation comprised answering a questionnaire with five five-level questions (very good, good, neither good nor bad, bad, very bad), each measuring five categories of experience: cooperation, information, examination/evaluation, treatment and overall evaluation of the consultation. (Questions presented in Table 3). The questions regarding information and treatment included the additional option ‘not applicable’. All of the questions were equally weighted, and a sum score was calculated. The primary outcome measurement was the difference between standard and video-assisted consultations in the sum score.

Additional analyses were done to support the evaluation of the professional quality of the consultation. The orthopaedic surgeon recorded the duration of the consultation as well as agreement on further action (follow-up consultation/discharge/referrals). The patients received a questionnaire three and 12 months after the last consultation to report events or complications, including any need for additional contact with health services as well as patient-reported outcome measures (EQ-5D-3L and EQ-VAS). Two postal reminders were sent, and an additional telephone call was placed to non-responders. The patients’ hospital medical records were screened for additional information relevant to the study. These included complications linked to the referred condition (reported or not by the patient); if referred for operation, whether operated as referred or not; total number of consultations for the actual disorder in the study and if they had been referred again for the same condition over the subsequent two years. The orthopaedic surgeon questionnaire after the video-assisted consultations included five additional five-level questions (very good, good, neither good nor bad, bad, very bad) regarding cooperation with other health workers, technical issues, previous experience with video-assisted consultations and expectations regarding a video-assisted consultation compared to a standard consultation before and after the conducted consultation.

The secondary endpoints were comprised of patient satisfaction and economic analyses, assessed via questionnaires given to the patients and specialists after each consultation and mailed to the patients three and 12 months after the last consultation in the study. The health economic outcomes and patient satisfaction will be reported in separate papers.

Baseline data were collected via a questionnaire that the patients completed immediately after the first consultation. This included demographic variables (age, gender, occupation, education), indicators used for measuring patient-reported outcomes, cause of consultation and experience with different specialist outpatient clinics. English translations of the questionnaires used in the study can be viewed in the Additional files 1, 2, 3 and 4.

### Sample size

The sample size calculation was based on the quality sum score assessed by the consulting physicians; the results indicated that we needed at least 191 patients in each group to achieve 90 % power to detect non-inferiority using a one-sided two-sample t-test, a standard deviation equal to 1.0 and a 5 % significance level. The margin of non-inferiority was set at 0.30, as a difference in sum score between the groups ≤ 0.3 was rated as not clinically relevant using a questionnaire with five-level questions (1–5).

### Randomisation

Randomisation of patients was performed via a password-protected, web-based randomisation database created by the Unit for Applied Clinical Research, Faculty of Medicine, Norwegian University of Science and Technology, Trondheim. It was a blocked randomisation of unknown size and stratified by municipality and age (≤18 and ≥ 65 in one group and 19–64 years of age in the other). Blinding was not applicable.

### Implementation

Some of the patients were referred directly to participate in the study by their General Practitioner (GP) or specialists at the hospital, but most of the eligible patients were contacted for inclusion after review (by a secretary or the corresponding author) of the hospitals’ waiting lists or evaluation of newly referred patients. Up to two invitation letters were sent by mail. The orthopaedic surgeon running the study did the final evaluation to ensure that each patient met the inclusion criteria; the same surgeon also performed the randomisation. The study patients were thereafter given a consultation appointment according to a planned schedule.

### Statistical methods

The baseline characteristics were presented as means (standard deviation) or numbers (percentages). Generalised estimating equations (GEE) were used to assess the differences between the intervention and the control group and to assess the non-inferiority of the intervention group. The exchangeable covariance structure was specified in the GEE models in order to control for two or more consultations for some of the participants. In additional models, we recoded the items regarding the evaluation of the consultation to very good (yes/no) and used GEE assuming a binomial distribution with a logit link function. The study design was based on the intention to treat principle, but the analyses of the primary outcome – the sum score – were not strictly by intention to treat principle, since 3.2 % of the randomised patients did not meet for a consultation (5.0 % in the control and 1.5 % in the intervention group). The ancillary results were presented as means (standard deviation) or numbers (percentages) and analysed using two-sample t-tests or chi-square tests, as appropriate. Statistical analyses were performed using STATA version 13.1 (StataCorp LP Texas, USA).

## Results

**Fig. 1:**
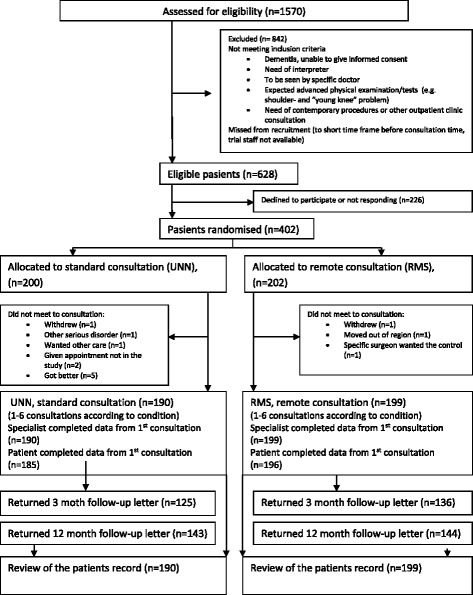
Flow diagram of the enrollment, allocation, follow- up and data collections points

Eligible patients from the four municipalities were recruited between November 2007 and August 2012 and were seen at the outpatient clinic at the first available slot after randomisation, or for follow-up patients, when scheduled. The last consultation in the study was conducted in October 2012. A review of the patient files was performed between May 2013 and October 2014. The baseline characteristics are shown in Table 2; they did not reveal any significant differences between the groups. Figure 1 shows the flow chart, including the data collection points. A total of 559 consultations (257 at UNN and 302 at RMC) from 389 patients (190 at UNN and 199 at RMC) were included. The specialists’ evaluation questionnaires were completed for all of the consultations (100 %); one consultation in each group missed all of the questions, forming the sum score (0.5 %). A total of 547 (98 %) of the patients completed the questionnaire (249 at UNN and 298 at RMC). One patient in each group did not attend their follow-up appointments due to other more serious disorders. A total of 125 (66 %) of the UNN-allocated patients versus 136 (68 %) of the RMC participants returned the 3-month questionnaire, and 143 (75 %) and 144 (73 %) returned the 12-month questionnaire. All 389 participating patients’ electronic medical records were reviewed as planned. Four patients from UNN and two from RMC died of other disorders within two years after their last consultation.

**Table 2:** Descriptive baseline characteristics from 1st consultation according to location ^a^

	UNN, standard consultation (*n* = 190)	RMC, video conference consultation (*n* = 199)
Males	75 (39)	82 (41)
Age, years	46.7 ± 24.9	48.8 ± 24.0
Age		
1-18 years	46 (24)	43 (22)
19-64 years	86 (45)	91 (46)
65-90 years	58 (31)	65(33)
The patient residential municipality		
Kvænangen	25 (13)	26 (13)
Nordreisa	82 (43)	90 (45)
Skjervøy	47 (25)	45 (23)
Kåfjord	36 (19)	38 (19)
Cause of consultation		
New referral	69 (36)	81 (41)
Control after elective surgery	25 (13)	22 (11)
Control after trauma surgery	33 (17)	35 (18)
Control after trauma, no surgery	55 (29)	50 (25)
Chronic disease	8 (4)	11 (6)
EQ-5D-3 L index (n = 165 + 178)^b^	0.70 ± 0.25	0.68 ± 0.26
EQ VAS 1–100 (*n* = 140 + 150)^b^	75 ± 18	73 ± 19
Patient assessment of own health in general; 5-leveled scale (*n* = 180 + 191)^b^	2.00 ± 0.83	2.05 ± 0.83
Employment status (n = 177 + 190)^b^		
Full time worker	45 (25)	56 (30)
Part time worker	23 (13)	20 (11)
Homemaker	12 (7)	19 (10)
Unemployed	2 (1)	2 (1)
Retired/disability benefit	55 (31)	61 (32)
Student/pupil	40 (23)	32 (17)
Education (*n* = 158 + 176)^b^		
Primary school	85 (54)	92 (52)
Secondary school	39 (25)	54 (31)
University	34 (21)	30(17)
Number of outpatient consultations last 6 months before 1st consult. (n = 180 + 188)^b^		
Only the actual consultation	109 (61)	128 (68)
2 to 3 times	64 (36)	52 (28)
4 times or more	7 (4)	8 (4)

*UNN* University Hospital of North Norway, *RMC* Regional Medical Centre

^a^ Values are mean ± SD or number (percent)

^b^ Number of item responses in UNN and RMC respectively

### Outcomes and estimation

The reasons for discharge from the study were as follows: patient did not need further follow-up (*n* = 216, RMC 113 [57 %]/UNN 103 [55 %]); patient was referred for surgery (*n* = 55, RMC 22 [11 %]/UNN 33 [17 %]); patient was referred to another outpatient clinic (*n* = 8, RMC 3 [2 %]/UNN 5 [3 %]); patient required further follow-up at the orthopaedic department for chronic conditions (*n* = 74, RMC 41 [21 %]/UNN 33 [17 %]); patient required follow-up with his or her own GP (*n* = 6, RMC 2 [1 %]/UNN 4 [2 %]); patient needed a consultation specific to the orthopaedic outpatient clinic at UNN (*n* = 27, RMC 16 [8 %]/UNN 11 [6 %]); patient was referred for admission to the ward (RMC 1 [0.5 %]/UNN 0 [0 %]) (*p* = 0.424). The reasons that 27 patients needed follow-up consultations specific to UNN (standard consultation) were as follows: the physician was not satisfied with the examination at the remote location (*n* = 3); patient needed removal of osteosynthesis implants (*n* = 13); patient needed diagnostic anaesthetic injection tests (*n* = 3); patient needed a CAT scan (*n* = 5); other causes (*n* = 3). Except for ‘not satisfactorily examined at the remote location’, these causes were equally distributed between both groups.

**Fig. 2:**
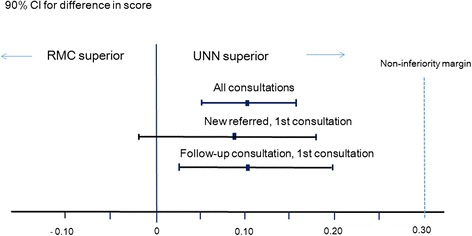
Observed treatment differences for video-assisted consultation (RMC) minus standard consultation (UNN) for sum-score of the specialist evaluation of the consultation. Blue dashed line = 0.3 non-inferiority margin, CI = Confidence interval

The primary outcome – the sum score of the orthopaedic surgeon’s evaluation – was significantly lower, in other words, ‘better’, at UNN compared to RMC (1.72 versus 1.82, *p* = 0.0030). However, the 90 % CI for the difference in score (0.05, 0.17) was within the non-inferiority margin (Fig. 2). Subgroup analyses restricted to the first consultation of newly referred patients (*n* = 150) and the first follow-up consultation of those who were not newly referred (*n* = 238) showed similar results with slightly wider CIs (−0.02, 0.18) and (0.03, 0.20), respectively. When the five different questions forming the sum score were assessed separately, the questions regarding how the orthopaedic surgeon evaluated the examination/evaluation of the patient and the overall evaluation of the consultation demonstrated significantly higher scores in the RMC group (Table 3).

**Table 3:** Orthopaedic surgeon’s evaluation of the consultation per allocation^a^

	UNN,	RMC, video	*p*-value^b^	*p*-value^c^
	standard consultation	conference consultation		
How well did you perceive the patient cooperated during the consultation? (254 + 299)^d^			*p* = 0.58	*p* = 0.75
Very good	95 (37)	105 (35)		
Good	157 (62)	190 (64)		
Neither good nor bad	2 (1)	3 (1)		
Bad	0 (0)	1 (0)		
Very bad	0 (0)	0 (0)		
How well could you evaluate/examine the patient? (243 + 290)^d^			*P* < 0.001	*P* < 0.001
Very good	98 (40)	57 (20)		
Good	144 (59)	225 (78)		
Neither good nor bad	1 (0)	7 (2)		
Bad	0 (0)	1 (0)		
Very bad	0 (0)	0 (0)		
How well could you treat the patient? (246 + 292)^d^			*p* = 0.068	*p* = 0.039
Very good	23 (16)	12 (7)		
Good	119 (83)	155 (91)		
Neither good nor bad	1 (1)	2 (1)		
Bad	0 (0)	1 (1)		
Very bad	1 (1)	0 (0)		
Other (not applicable)	102	122		
How well could you inform the patient? (254 + 298)^d^			*p* = 0.106	*p* = 0.28
Very good	54 (22)	50 (17)		
Good	191 (77)	233 (79)		
Neither good nor bad	4 (2)	12 (4)		
Bad	0 (0)	0 (0)		
Very bad	0 (0)	0 (0)		
Other (too young)	5	3		
Overall how well could you assess/treat/checking the patient? (254 + 293) ^d^			*p* = 0.0047	*p* = 0.040
Very good	56 (22)	43 (15)		
Good	198 (78)	242 (83)		
Neither good nor bad	0 (0)	7 (2)		
Bad	0 (0)	1 (0)		
Very bad	0 (0)	0 (0)		
Sum score, mean(SD)	1.72 ± 0.38	1.82 ± 0.38	*p* = 0.0030	NA

*UNN* University Hospital of North Norway, *RMC* Regional Medical Centre

^a^ Values are number (percent) or mean ± SD

^b^ Test for equality between UNN and RMC using generalised estimating equations (GEE)

^c^ Test for equality between UNN and RMC using GEE with a logit link function and a binary response very god (yes/no)

^d^ Number of item response in UNN and RMC respectively

There were a few missing values in the five questions forming the sum score from 6 up to 26 (1.1–4.7 %). A sensitivity analysis, in which the missing values were replaced with the highest score in the intervention group and the lowest score in the control group, still demonstrated a difference in sum score that was within the non-inferiority margin (90 % CI 0.14–0.27).

### Ancillary analyses

Additional analyses are shown in Table 4. The mean consultation duration was not significantly different between the groups (*p* = 0.60). In the subgroup analyses restricted to patients who required casting, we observed a significantly longer mean consultation time in the RMC group (29.0 min) compared to the UNN group (22.6 min, *p* = 0.0063). Casting was performed in 11 % of the consultations. All of the patients at the RMC underwent their planned operation. In the UNN group, two patients were not operated on due to the occurrence of other serious disorders, four patients improved during the waiting time and did not need the planned surgery and one did not appear for an unknown reason. There were no significant differences in the number of operated patients between the two groups (*p* = 0.432). Of the 190 patients allocated to UNN, 147 had one consultation, 27 had two, 11 had three, three had four, one had five and one patient had six consultations before discharge from the study. Of the 199 patients allocated to the RMC, 135 had one consultation, 39 had two, 15 had three, seven had four, two had five and one had six consultations. There was a tendency toward more consultations in the RMC group, but this was not statistically significant (*p* = 0.057). Also, the subgroup analyses of the number of consultations per patient according to the cause of the consultation did not demonstrate any significant differences. The patients who had their appointment at the RMC were not more likely to be referred again within two years for the same disorder (*p* = 0.858). Furthermore, no significant difference was observed in the subgroup of ‘discharged patients’ (i.e. in those who were not referred for operation, a standard consultation or any follow-up appointment for chronic disorders with the orthopaedic department within six months). The patient-reported outcome measure at three and 12 months and the change from the baseline to 12 months did not demonstrate any difference between the two groups. This will be analysed in a separate paper.

**Table 4:** Ancillary results according to location^a^

	UNN, standard consultation (*n* = 190)	RMC, video conference consultation (*n* = 199)	*P*- value^**^
Consultation durations, minutes^b^	20.9 ± 7.47	20.5 ± 8.9	0.603
Operation			
Referred to surgery	33 (17 %)	22 (11 %)	0.074
Operated	26 (14 %)	22(11 %)	0.431
Referred again within 2 years			
Overall (n = 190 + 199)	19 (10 %)	21 (11 %)	0.858
Among “discharged patient” (n = 145 + 159)^c^	12 (8 %)	18 (11 %)	0.373
Number of consultations per included			
Overall (n = 190 + 199)	1.35 ± 0.78	1.52 ± 0.91	0.057
New referred^d^ (n = 69 + 81)	1.06 ± 0.29	1.17 ± 0.44	0.067
Control patients^e^ (n = 121 + 118)	1.52 ± 0.91	1.75 ± 1.01	0.071
Complication			
Overall (n = 190 + 199)^g^	40 (21 %)	33 (17 %)	0.259
Patient reported at 3 month,(n = 109 + 119)^f^	15 (14 %)	16 (13 %)	0.095
Patient reported at 12 month, (n = 132 + 133)^f^	23 (17 %)	14 (11 %)	0.105

*UNN* University Hospital of North Norway, *RMC* Regional Medical Centre

^a^ Values are mean ± SD and number (percent)

^b^ 553 consultations, missing data: 4 of 257 in UNN and 3 of 302 in RMC group

^c^ Patient with no appointment at orthopedic department within 6 month for the actual disorder, presented according to location. (Patient neither referred to operation nor to a required standard consultation or follow-up for chronic disorder)

^d^ One patient in each group did not meet to follow up consultation

^e^ Cause of consultation – control after elective surgery, trauma or chronic diseases

^f^ Denominator/number differs due to non-item response, presented according to location

^g^ Evaluation of the patient’s records and patient reported at 3 and 12 months, presented according to location

^**^ *P*-value calculated with t-test or chi square test when appropriate

### The telemedicine consultation

For the video-assisted consultations, the orthopaedic surgeon evaluated the cooperation with other health workers as ‘very good’ (99 %) and ‘good’ (1 %) and the technical performance as ‘very good’ (14 %), ‘good’ (78 %), ‘neither good nor poor’ (7 %) and ‘poor’ (<1 %). There was no change in the orthopaedic surgeons’ evaluation of a video-assisted consultation compared to a standard consultation before and after the actual consultation, which were evaluated as equal (98–99 %). All of the video-assisted consultations were conducted as planned. Due to technical trouble, 17 consultations were delayed – two subsequent consultations for 75 and 60 min, the rest for 17 min (mean).

## Discussion

The main finding in our study is that the orthopaedic surgeon evaluated the video-assisted consultations as not being inferior to the standard consultations. The sum score was significantly lower in the control group compared to the intervention group, but the difference was within the non-inferiority margin. The difference in sum score was 0.1 on a scale from 1 to 5, which is lower than the assumed accepted difference of clinical relevance. A total of 98 % of the remote consultations versus 99 % of the standard consultations were evaluated as ‘good’ or ‘very good’ for all of the questions in the questionnaire, except for the question regard information to the patient which for 96 % of the consultations at RMC were evaluated as ‘good’ or ‘very good’. X-rays are an important part of an orthopaedic consultation. In our study, X-rays were performed immediately prior to the consultations in 88 % (UNN) and 87 % (RMC) of the cases. This might contribute to the orthopaedic surgeons’ positive evaluations. At an orthopaedic consultation, it is important to reach a conclusion for a further treatment plan based on the patient’s history, the clinical examination/evaluation and any additional tests or investigations (mainly X-rays). Therefore, it is expected that a consultation without the possibility of physically examining the patient directly will be evaluated as less optimal than a standard consultation. This could explant the significant difference in evaluation of question regarding evaluation/examination of the patient. The overall question of how well the orthopaedic surgeon could assess/treat/check the patient is also influenced by the latter.

Due to the lack of a standard validated questionnaire for the orthopaedic surgeons’ evaluation of the consultations, we created one. The five questions relevance were evaluated by eight, not in the study engaged, orthopaedic surgeons, item content validity index, CVI = 0.976, calculated and reported as recommended by Polit and Beck [19]. All of the questions were related to assessment, which could be affected by the different consultation situations. Others have used similar questions. For example, Brennan et al., who evaluated emergency physicians’ ability to use telemedicine to evaluate and treat patients with pre-selected chief complaints in an emergency department, reported a mean of 3.8 (1 = not very satisfied, 5 = very satisfied) in the physicians’ comfort level in making diagnoses and performing treatment in the telemedicine group. They did not report any mean in the control group or p-values, but they concluded that telemedicine was a satisfactory technique for the chosen group of patients [5]. A similar result was reported by Wan et al. They evaluated the feasibility of remote consultation for pain management, orthopaedics and general surgery using telemedicine. They had a mean score of 3.6 for the physicians’ satisfaction with seeing the patient via videoconference [20]. Aarnio et al. found that 23 out of 29 (six missing) orthopaedic surgeons responded with ‘good’ or ‘very good’ as their level of overall satisfaction with teleconsultations, and 20 evaluated the physical examination with aid as ‘good’ or ‘very good’ [8]. In another study regarding remote surgical consultations by videoconference, Aarnio et al. demonstrated that 92 % of the consulting surgeons fully agreed that their decisions were as good as they would have been in a usual outpatient clinic consultation [21].

In this study, we did not find any serious events related to the mode of consultation. This finding is strengthened by the fact that our institution is the only hospital in this region, thereby allowing us to discover serious events that the participants do not report, as long as these resulted in contact with the hospital. The patient-reported complications included a wide variety of causes, many of which were not related to the treatment or patient evaluation at the consultations. The complications, evaluated based on the patients’ reports, and total complications, which also include complications revealed from the patients’ medical records, were not different between the two groups.

Because of the lack of a standard questionnaire for measuring orthopaedic surgeons’ satisfaction of consultations, we performed additional analyses to support the evaluation of the quality of care of the consultation. We did not find any significant difference between the two groups concerning referral to operation, regardless of whether the planned operations were performed or not. This was also the case when the analysis was restricted to the new referred patients (data not presented), which is in conjunction with the findings of another follow-up study on videoconferencing with orthopaedic outpatients [9].

Another important finding in our study is that the mean consultation duration was not significantly different between the groups. This is in contrast to what others have reported, where the duration of telemedicine consultations was significantly longer than that of standard consultations [4]. Our data does not give a clear explanation for this finding, although our consultations’ duration of 20 min generally was longer [8, 22]. The scheduled duration for each consultation (including consultation, documentation and study registration) was 30 min, which may have influenced the overall amount of time. Another factor could be that all of the consultations in our study were scheduled. Urgent consultations, which represented the largest proportion of consultations in other studies, were not included [4, 10, 23].

One could expect that if the patients were not satisfied with the outcome of the consultation, they would be more likely to be referred again if they still had problems or pain. We did not find any difference between the groups regarding re-referrals, or when analysing subgroups according to different causes of inclusion or how they were discharged from the study. These findings support that, in our study, videoconference consultations are not inferior to standard care. To our knowledge, others have not reported this.

Even if there was a tendency toward a higher number of consultations per patient in the video-assisted group, the difference between the two groups was not significant. After the first consultation, 32 % of the patients in the RMC group were discharged compared to 36 % in the UNN group (*p* = 0.389). Wallace et al. reported that patients in the virtual outreach group were offered follow-up appointments to a larger degree compared to patients receiving standard consultations, especially orthopaedic and ear, nose and throat (ENT) patients [24]. Another study reported that a significantly higher proportion of patients assessed by an emergency medicine specialist using telemedicine were offered a follow-up consultation compared to patients assessed by an on-site emergency medicine specialist [4]. One possible explanation for this difference could be our thorough evaluation of the participants’ orthopaedic condition before their inclusion in the study. For example, we did not include the first visit for emergency patients and excluded patients with an expected need for advanced clinical examination or treatment. Two of the three patients who were not satisfactorily evaluated at RMC had a combination of back and hip pain. Another study has also reported inadequate assessment of patient histories that present with back problems at telemedicine consultations [6].

Our telemedicine approach might be improved if it was an option to have another trained health worker together with the patient at the remote site than the trained nurses used in our study. For example, in a further study on video assisted remote consultations for orthopaedic patients it could be tested whether the possibility to have a physiotherapist together with the patient could increase the potential for examining/testing the patients, and thus both increase the quality of the telemedicine consultations and expand its use to a wider range of patients.

## Conclusions

This study found that it was safe to offer video-assisted remote consultations for selected orthopaedic patients. The strengths of this study are that is was conducted in a real-life clinical setting. We did not find any serious events related to the mode of consultation. Further assessments of the economic aspects and patient satisfaction are needed before we recommend a wider application.

## Additional files

Additional file 1: Questionnaire for UNN allocated patients, English translation. English translation of questionnaire for patients allocated to standard consultation, immediately after the consultation. (DOCX 24 kb) Additional file 2: Questionnaire for RMS allocated patients, English translation. English translation of questionnaire for patients allocated to telemedicine consultation, immediately after the consultation. (DOCX 30 kb) Additional file 3: Questionnaire for the orthopaedic surgeon performing the consultation, English translation. English translation of questionnaire for the orthopaedic surgeon performing the consultation, immediately after the consultation. (DOCX 16 kb) Additional file 4: Follow up questionnaire for patients participating in the study, at 3 and 12 months, English translation. English translation of questionnaire for the participating patients at 3 and 12 months follow up. (DOCX 27 kb)

## Abbreviations

CI: Confidence interval
CVI: Content validity index
GEE: Generalized estimating equations
RCT: Randomised controlled trial
RMC: Regional Medical Centre
UNN: University Hospital North Norway
