# Supplementary material for: Quality of care for remote orthopaedic consultations using telemedicine: a randomised controlled trial
Source: BMC Health Serv Res. 2016 Sep 8;16(1):483. doi: 10.1186/s12913-016-1717-7 (PMC5017045; doi:10.1186/s12913-016-1717-7)
Supplement: Additional file 1: — Questionnaire for UNN allocated patients, English translation. English translation of questionnaire for patients allocated to standard consultation, immediately after the consultation. (DOCX 24 kb) [file 12913_2016_1717_MOESM1_ESM.docx]

“Teleorthopaedic study”

**Questionnaire number 2 to patient who has been to consultation at the orthopaedic outpatient clinic at UNN (Standard consultation) or Sonjatun (Telemedicine)**

**after: _ (3 )and _ (12) months.**

1. Date of completing the questionnaire dd mm yyyyy
2. How long time since operation or injury?

00 (months)

0 Not applicable

1. How useful do you think the surgery has been for you?

0 – Very useful

0 – Little useful

0 – No useful

0 – Got worse

0 – Not applicable

1. In total, how satisfied or unsatisfied are you with the consultation at the orthopaedic outpatient clinic (UNN or Sonjatun)?

0 – Very satisfied

0 – Quite satisfied

0 – Neither satisfied nor unsatisfied

0 – Quite unsatisfied

0 – Very unsatisfied

1. Has any complication after the treatment occurred?

0 – Yes

0 – No

If yes, who has treated you (one or more ticks)?

0 – No one

0 – My general practitioner

0 – Emergency doctor

0 – Home care service

0 – Outpatient clinic at the hospital

0 – Admitted at an infirmary/nursing home

0 – Admitted in hospital

How serious do you think your complication(s) is/are?

0 – Very serious

0 – Serious

0 – Not serious

What kind of complications (one or more ticks)

0 – Bleeding

0 – To tight cast or other complication (The cast has been changed because of this)

0 – Infection

0 – Increased pain

0 – Overseen injury

0 – Others (specify)………………………………………

1. How do you in general consider your own health to be?

0 – Very good

0 – Good

0 – Neither good nor bad

0 – Bad

0 – Very bad

1. What is your main occupation/activity? (Tick one)

0 – Full time work

0 – Part time work

0 – Housekeeping

0 – Unemployed

0 – Retired/benefit recipient

0 – Student/pupil

1. Are you on a sick leave? (one or more ticks)

0 – Yes,

0 – according actual disorder

0 – other disorder

0 – No

How long have you received sickness benefit for actual disorder _ _ _ weeks

1. Have you applied for compensation to the national insurance/other insurance because of your health problem?

0 – Yes

0 – No

0 – Plan to apply

0 – Already approved

1. Who completed the questionnaire?

0 – Myself

0 – Guardian because the patient is too young

0 – With help from companion

0 – With help from personnel at the outpatient clinic

0 – The companion since the patient is unable to do it

**Description of your health status**

Mark the statement that best fits your state of health today by ticking once in one of the five groups below:

1. Mobility

0 – I have no problems in walking about

0 – I have little problems in walking about

0 – I am confined to bed

2. Self-care

0 – I have no problems with self-care

0 – I have some problems washing or dressing myself

0 – I am unable to wash or dress myself

3. Usual activities (e.g. work, study, housework, family or leisure activities)

0 – I have no problems with performing my usual activities

0 – I have some problems with performing my usual activities

0 – I am unable to perform my usual activities

4. Pain and discomfort

0 – I have no pain or discomfort

0 – I have moderate pain or discomfort

0 – I have extreme pain or discomfort

5. Anxiety and depression

0 – I am not anxious or depressed

0 – I am moderately anxious or depressed

0 – I am extremely anxious or depressed

To allow you to show us how good or bad your state of health is we have made a scale (almost like a thermometer) where the best state of health you can imagine is marked 100 and the worst 0. We ask you to show your state of health by drawing a line from the box below to the point on the scale that best fits your state of health.

Best imaginable health state


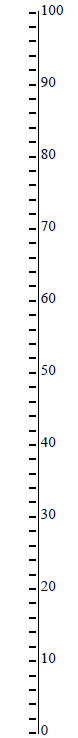


Your own health

state today

Worst imaginable health state
