# Supplementary material for: Quality of care for remote orthopaedic consultations using telemedicine: a randomised controlled trial
Source: BMC Health Serv Res. 2016 Sep 8;16(1):483. doi: 10.1186/s12913-016-1717-7 (PMC5017045; doi:10.1186/s12913-016-1717-7)
Supplement: Additional file 3: — Questionnaire for the orthopaedic surgeon performing the consultation, English translation. English translation of questionnaire for the orthopaedic surgeon performing the consultation, immediately after the consultation. (DOCX 16 kb) [file 12913_2016_1717_MOESM3_ESM.docx]

“Teleorthopaedic study”

**Questionnaire to the orthopaedic surgeon conducting the consultation at UNN (standard consultation) or Sonjatun (telemedicine)**

1. Date of consultation dd mm yyyy
2. Telemedicine consultation 0 ;

Standard consultation 0

1. Cause of consultation?

0 – New referral

0 – Follow-up after elective surgery

0 – Follow-up after trauma, surgery

0 – Follow-up after trauma, no surgery

0 – Chronic disease

1. Has the consultation taken place?

0 – Yes

0 – No

If not, reason………………….

1. Activity during the consultation? (More than one tick possible)

0 – Removal of a cast

0 – Casting

0 – X-ray been taken

0 – “Examination of the patient”

0 – Sick leave formula completed

0 – Prescription

0 – Other schemes

1. What is the patient’s main occupation/activity? (Tick one)

0 – Full time work; 0 – heavy manual working, 0 – office work

0 – Part time work

0 – Housekeeping

0 – Unemployed

0 – Retired/benefit recipient

0 – Student/pupil

1. Is the patient on a sick leave? (one or more tick)

0 – Yes according to actual disorder; _ _ %

0 – other disorder

How long have the patient received sickness benefit for actual disorder 000 weeks

1. Who were present at the consultation (total number – both UNN and Sonjatun during the telemedicine consultation)

Nurse _ _

Physiotherapist _ _

Doctor/orthopaedic surgeon _ _

1. Did the patient suffer from a medical condition which gave indication for special transport?

0 – No

0 – Yes

0 – Taxi

0 – Ambulance

1. Did the patient suffer from a medical condition which gave indication for companion?

0 – No

0 – Yes

1. Time spent for the consultation (the patient present)

000 minutes

1. Further plan/follow-up

0 – follow-up (UNN/Sonjatun)

0 – Further investigation

0 – Further treatment (f. ex fracture healing)

0 – Scheduled follow-up (chronic disease/condition)

0 – Orthopaedic outpatient clinic UNN; Cause……………………………..

0 – Referred to admission in hospital

0 – Referred to day surgery

0 – Referred to other outpatient clinic/ hospital/health service

0 – Follow up by general practitioner

0 – Discharge (i.e. no planned follow up)

1. How well did you perceive the patient cooperated during the consultation?

0 – Very good

0 – Good

0 – Neither good nor bad

0 – Bad

0 – Very bad

1. How well could you evaluate/examine the patient?

0 – Very good

0 – Good

0 – Neither good nor bad

0 – Bad

0 – Very bad

1. How well could you treat the patient?

0 – Very good

0 – Good

0 – Neither good nor bad

0 – Bad

0 – Very bad

0 – Not applicable

1. How well could you inform the patient?

0 – Very good

0 – Good

0 – Neither good nor bad

0 – Bad

0 – Very bad

0 – Not applicable (too young patient) ……………………………………….

1. Overall, how well could you assess/treat/check the patient?

0 – Very good

0 – Good

0 – Neither good nor bad

0 – Bad

0 – Very bad

1. How did you experience the cooperation with the other personnel at the outpatient clinic?

0 – Very good

0 – Good

0 – Neither good nor bad

0 – Bad

0 – Very bad

1. If present, how well could you inform the companion about the patient’s condition?

0 – Not applicable

0 – Very good

0 – Good

0 – Neither good nor bad

0 – Bad

0 – Very bad

**Additional for Telemedicine consultation - Sonjatun**

1. How well did it work technically

0 – Very good

0 – Good

0 – Neither good nor bad

0 – Bad

0 – Very bad

0 – reason……………………………………………

1. In how many telemedicine consultations have you taken part earlier?

0 – No

0 – 1 to 5

0 – 5 to 15

0 – 15 to 30

0 – More than 30

1. *Before this consultation* how did you think a telemedicine consultation would be compared to a standard consultation

0 – Much better

0 – Better

0 – Just as good

0 – Worse

0 – Much worse

1. Now, compared to a standard consultation how do you think a telemedicine consultation is?

0 – Much better

0 – Better

0 – Just as good

0 – Worse

0 – Much worse
