# Supplementary material for: Quality of care for remote orthopaedic consultations using telemedicine: a randomised controlled trial
Source: BMC Health Serv Res. 2016 Sep 8;16(1):483. doi: 10.1186/s12913-016-1717-7 (PMC5017045; doi:10.1186/s12913-016-1717-7)
Supplement: Additional file 4: — Follow up questionnaire for patients participating in the study, at 3 and 12 months, English translation. English translation of questionnaire for the participating patients at 3 and 12 months follow up. (DOCX 27 kb) [file 12913_2016_1717_MOESM4_ESM.docx]

“Teleorthopaedic study”

**Questionnaire concerning patient experience with the orthopaedic consultation at UNN (Standard consultation)**

**Before (you came to) the consultation at UNN**

1. Regarding the time from referral till you got an appointment, how acceptable was the waiting time?

0 – I did not wait, I came to a scheduled follow-up consultation

0 – Completely acceptable

0 – Acceptable

0 – Neither acceptable nor unacceptable

0 – Unacceptable

0 – Very unacceptable

1. How important do you think this consultation at the outpatient clinic is?

0 – Very important

0 – Somewhat important

0 – Not important

1. How long travel time did you need from home to UNN?

0 – less than 1 hour

0 – About 1 to 2 hours

0 – About 3 to 4 hours

0 – About 5 to 8 hours

0 – More than 8 hours

1. How many kilometre is it?

0 – Less than 150 km

0 – 151 – 200 km

0 – 201 – 250 km

0 – 251 – 300 km

0 – 301 – 350 km

0 – More than 350 km

1. Did you have to stay overnight because of the travelling distance?

0 – Yes

0 – No

1. How did you get to the outpatient clinic (more than one tick possible)?
2. – private car

0 – Taxi

0 – Bus

0 – Ferry

0 - Catamaran

0 – Airplane

0 – Other option ………..

1. Did you have a companion?

0 – No

0 – Yes

0 – Family member

0 - Homecare or Companion service

0 - Acquaintance

**Reception at - UNN**

1. How satisfied were you with the way you were received?

0 – Very satisfied

0 – Satisfied

0 – Neither satisfied nor unsatisfied

0 – Unsatisfied

0 – Very unsatisfied

1. How long time did you have to wait before you were called?

0 – Less than 15 min

0 – Waited 15 to 30 min

0 – Waited 30 min to 1 hour

0 – Waited more than 1 hour

**Consultation**

1. Did you get an appointment with a doctor you have seen earlier?

0 – Yes

0 – No

0 – Have not been to a consultation before

1. According to your experience, how well did the personnel at the outpatient clinic cooperate regarding your consultation?

0 – Very good

0 – Good

0 – Neither good nor bad

0 – Bad

0 – Very bad

1. Do you think it was enough time scheduled for talk during the consultation?

0 – Yes

0 – No

1. Do you think your doctor was *well prepared* for your actual consultation?

0 – Very good

0 – Good

0 – Neither good nor bad

0 – Bad

0 – Very bad

1. Did the doctor talk to you in an understandable way?

0 – Very good

0 – Good

0 – Neither good nor bad

0 – Bad

0 – Very bad

1. How good did you assess the doctor’s medical skill to be?

0 – Very good

0 – Good

0 – Neither good nor bad

0 – Bad

0 – Very bad

1. Were you met with courtesy and respect?

0 – Yes very good

0 – Yes, good

0 – Neither good nor bad

0 – No, bad

0 – No, very bad

1. Did you get the opportunity to tell all what you thought was important about your condition?

0 – Yes, every thing

0 – Mostly

0 – Sufficient

0 – Little

0 – Nothing

1. Did you get no know what you thought was necessary concerning results of tests and examinations during the consultation?

0 – Yes, every thing

0 – Mostly

0 – Sufficient

0 – Little

0 – Nothing

0 – There were no tests or examinations

1. Did you get to know what you should contribute by own efforts after the consultation?

0 – Yes

0 – To some extent

0 – No

1. Did you get to know how your condition would change in the immediate future?

0 – Yes

0 – To some extent

0 – No

1. If you wanted to, could you take part in the further assessment and treatment of your condition?

0 – Did not want

0 – Yes

0 – To some extent

0 – No

0 – Not applicable

1. Have you heard of a telemedicine consultation before?

0 – Yes

0 – No

1. Have you earlier been to a telemedicine consultation?

0 – No

0 – One time

0 – More times

1. How did you think a telemedicine consultation is compared to a standard consultation?

0 – Much better

0 – Better

0 – Just as good

0 – Worse

0 – Much worse

0 – Do not have any opinion

1. If you should need another consultation, where do you want the next consultation?

0 – at UNN

0 – at Sonjatun

1. How well fit the following statements for your preference?

I want the shortest traveling time to and from the consultation:

0 – Very high degree

0 – High degree

0 – Some degree

0 – Little degree

0 – Not at all

I want to combine the consultation with other activities

0 – Very high degree

0 – High degree

0 – Some degree

0 – Little degree

0 – Not at all

I want to support a local offer:

0 – Very high degree

0 – High degree

0 – Some degree

0 – Little degree

0 – Not at all

I want to meet the specialist directly:

0 – Very high degree

0 – High degree

0 – Some degree

0 – Little degree

0 – Not at all

1. In total, how satisfied or unsatisfied are you with the consultation at UNN

0 – Very satisfied

0 – Quite satisfied

0 – Neither satisfied nor unsatisfied

0 – quite unsatisfied

0 – Very unsatisfied

**Background questions**

1. Sex and age

0 – man

0 – woman

Age _ _ years

1. Is Norwegian your mother tongue?

0 – Yes

0 – No

1. How many times have you been to an outpatient clinic the last 6 months?

0 – Only this time

0 – 2 to 3 times

0 – 4 or more times

1. How do you in general consider your own health to be?

0 – Very good

0 – Good

0 – Neither good nor bad

0 – Bad

0 – Very bad

1. What is the highest level of education you have completed? (Tick one)

0 – Primary, 1 – 2 years secondary school

0 – Vocational school

0 – High secondary school (A-level)

0 – College/university less than 4 years

0 – College/university 4 years or more

1. What is your main occupation/activity? (Tick one)

0 – Full time work

0 – Part time work

0 – Housekeeping

0 – Unemployed

0 – Retired/benefit recipient

0 – Student/pupil

1. Are you on a sick leave? (one or more ticks)

0 – Yes,

0 – according to actual disorder

0 – other disorder

0 – No

For how long have you received sickness benefit for actual disorder: _ _ _ weeks

1. What was the house holds total taxable income last years? Include income from work, social benefits and similar.

0 – Less than 125 000 NOK

0 – 125 000 - 200 000 NOK

0 – 201 000 - 300 000 NOK

0 – 301 000 - 400 000 NOK

0 – 401 000 - 550 000 NOK

0 – 551 000 - 700 000 NOK

0 – 701 000 - 850 000 NOK

0 – Over 850 000 NOK

1. Who do you live with? (tick for each question and give the number)

Spouse/cohabitant 0 – Yes, 0 – No

Other persons older than 18 years 0 – Yes, 0 – No, Number 00

Persons younger than 18 years 0 – Yes, 0 – No, Number 00

1. Have you applied for compensation to the national insurance/other insurance because of your actual health problem?

0 – Yes

0 – No

0 – Plan to apply

0 – Already approved

1. Who completed the questionnaire?

0 – Myself

0 – Guardian because the patient is too young

0 – With help from companion

0 – With help from personnel at the outpatient clinic

0 – The companion since the patient is unable to do it

**Description of your health status**

Mark the statement that best fits your state of health today by ticking once in one of the five groups below:

1. Mobility

0 – I have no problems in walking about

0 – I have little problems in walking about

0 – I am confined to bed

2. Self-care

0 – I have no problems with self-care

0 – I have some problems washing or dressing myself

0 – I am unable to wash or dress myself

3. Usual activities (e.g. work, study, housework, family or leisure activities)

0 – I have no problems with performing my usual activities

0 – I have some problems with performing my usual activities

0 – I am unable to perform my usual activities

4. Pain and discomfort

0 – I have no pain or discomfort

0 – I have moderate pain or discomfort

0 – I have extreme pain or discomfort

5. Anxiety and depression

0 – I am not anxious or depressed

0 – I am moderately anxious or depressed

0 – I am extremely anxious or depressed

To allow you to show us how good or bad your state of health is we have made a scale (almost like a thermometer) where the best state of health you can imagine is marked 100 and the worst 0. We ask you to show your state of health by drawing a line from the box below to the point on the scale that best fits your state of health.

Best imaginable health state


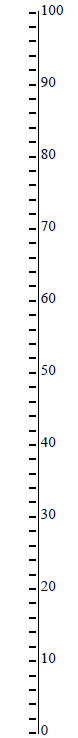


Your own health

state today

Worst imaginable health state
